# Staying active, staying sharp: the relationship between physical activity and health-related quality of life for people living with cognitive impairment

**DOI:** 10.1007/s11136-025-03910-5

**Published:** 2025-02-08

**Authors:** Rezwanul Haque, Khorshed Alam, Jeff Gow, Christine Neville, Syed Afroz Keramat

**Affiliations:** 1 School of Business, University of Southern Queensland, Toowoomba, QLD 4350 Australia; 2 Centre for Health Research, University of Southern Queensland, Toowoomba, QLD 4350 Australia; 3 School of Accounting, Economics and Finance, University of KwaZulu-Natal, Durban, 4001 South Africa; 4 School of Nursing and Midwifery, University of Southern Queensland, Toowoomba, QLD 4350 Australia; 5 Centre for Health Services Research, Faculty of medicine, The University of Queensland, Brisbane, QLD 4006 Australia

**Keywords:** Australia, Cognitive impairment, Health-related quality of life (HRQoL), Physical activity

## Abstract

**Background:**

Physical inactivity is a major global health concern and has been identified as a risk factor for cognitive impairment. In Australia, the long-term relationship between physical activity and health-related quality of life (HRQoL) in individuals with cognitive impairment remains under researched. This study aims to address this knowledge gap by using data from a population-based longitudinal study.

**Methods:**

We used data from two waves (wave 12 [2012] and wave 16 [2016]) of the Household, Income and Labour Dynamics in Australia (HILDA) Survey. Our final analytic sample consisted of 1,168 person-year observations from 985 unique individuals. To investigate the association between physical activity and HRQoL, we employed random-effects Generalized Least Squares (GLS) model.

**Results:**

We found that participants engaging in physical activity, < 1 to 3 times per week, showed significant positive associations with the Physical Component Summary (PCS) score [β = 4.41, Standard Error (SE) = 0.68], Mental Component Summary (MCS) score (β = 2.55, SE = 0.74), and SF-6D utility value (β = 0.05, SE = 0.007) compared to those who did not perform any physical activity. Similarly, participants who engaged in physical activity more than three times per week to every day had notably higher scores in PCS (β = 7.28, SE = 0.82), MCS (β = 4.10, SE = 0.84), and SF-6D utility values (β = 0.07, SE = 0.009).

**Conclusion:**

There is clear evidence that performing physical activity is positively associated with improved HRQoL in people with cognitive impairment. Our findings underscore the critical role of public health initiatives, such as health education and community-based programs, in promoting physical activity to enhance the HRQoL of older Australians living with cognitive impairment.

**Supplementary Information:**

The online version contains supplementary material available at 10.1007/s11136-025-03910-5.

## Introduction

The rising life expectancy, mostly driven by advancements in medical technology, is contributing to a growing global population of older adults aged 65 years and over [1, 2]. By 2066, the proportion of Australians aged 65 years or over is forecasted to rise to between 21% and 23%, a notable jump from the 16% recorded in 2020 [3]. Older adults are at a higher risk of experiencing cognitive decline, impacting their thinking, memory, concentration, and other brain functions [4]. Cognitive impairment, varying from mild to severe, is a major factor contributing to dependence and disability among older adults [5]. Mild cognitive impairment (MCI) is a condition whereby a person’s cognitive function is below normal, although they do not meet the criteria for dementia [6, 7]. People with MCI possess a significantly elevated risk of progressing to dementia relative to the general population [8], with an annual progression rate estimated between 10 and 15% [9, 10]. According to a recent meta-analysis, approximately 15% of community-dwelling adults aged 50 years and over are affected by MCI globally [11]. The estimated rate of cognitive impairment among Australians aged 65 years and older ranges from 7.7% to 33.3% [12, 13]. While currently no curative treatments exist for cognitive impairment related to dementia [14], non-pharmaceutical approaches remain a cornerstone of treatment for older adults experiencing cognitive impairment [8]. According to the World Health Organization, non-pharmacological interventions are recommended as the primary strategy for managing dementia symptoms and improving the well-being and quality of life of people living with dementia [15].

People with cognitive impairment have a higher propensity for physically inactive lifestyle [16], which may subsequently increase their risk of developing dementia [17] and aggravate cognitive decline [18]. Moreover, chronic illnesses along with physical inactivity can have a detrimental impact on people’s health and well-being, resulting in decreased overall health, reduced physical performance, and lower health-related quality of life (HRQoL) [19, 20]. HRQoL is a key component of assessing the health and well-being of older adults, providing valuable insights into their overall quality of life during the ageing process [21], and can inform decisions about preventing and treating illnesses [22].

Regular physical activity has many positive effects on health, both physical and mental [23]. Existing evidence based on cross-sectional [24–26] and longitudinal data [27, 28] have consistently demonstrated that people who engage in recommended levels of physical activity have better HRQoL compared to those who are less active, particularly among older population. Additionally, a recent systematic review emphasized that engaging in frequent physical activity can benefit older adults by enhancing their functional mobility, independence, reducing anxiety, improving balance, and fostering better social interactions [29]. While some longitudinal studies in Australia have shown a positive relationship between physical activity and HRQoL, these studies have primarily focused on the general population [30] or older people with disabilities [31]. Existing research suggests that physical activity is a strong protective factor against cognitive decline and can have beneficial effects on both cognitive and non-cognitive functions in people with cognitive impairment and dementia [6, 14, 32]. For instance, a meta-analysis of 18 randomized controlled trials suggests that physical activity improved cognitive function and quality of life for individuals living with dementia [33]. The study also revealed a positive effect of physical activity interventions on cognitive function across different population groups [33]. The primary analysis showed a substantial positive effect with a standardized mean difference (SMD) of 0.42 (95% CI: 0.23, 0.62). In individuals with Alzheimer’s disease (AD), physical activity interventions also demonstrated a beneficial effects (SMD = 0.38, 95% CI: 0.09, 0.66). A positive effect was also observed in a broader group including individuals with AD or a non-AD dementia diagnosis (SMD = 0.47, 95% CI: 0.14, 0.80). These findings consistently support the notion that physical activity interventions can have a positive impact on cognitive function in individuals living with cognitive impairment and dementia.

Physical activity is an effective intervention for promoting brain health in older adults, as it offers a low-cost and low-risk approach to improve cognitive function [34]. People with disabilities often benefit more from exercise than people without disabilities, especially for overall health and well-being [33, 35]. According to the World Health Organisation, older people may mitigate cognitive decline by performing a minimum of 150 min of moderate-intensity or 75 min of vigorous-intensity aerobic activity, along with strength training per week [36]. Despite numerous efforts to encourage physical activity among Australians, only 18% of the adult population met the recommended guidelines in 2022 [37].

The beneficial effects of physical activity are well-established, particularly in terms of preventing and managing cognitive decline. However, the relationship between physical activity and HRQoL among older Australians with cognitive impairment has not been thoroughly investigated. A significant gap exists in Australian research regarding the relationship between physical activity levels and HRQoL specifically among older adults with cognitive impairment. To address this gap, we explored the relationship between physical activity and HRQoL using a nationally representative longitudinal data. Insights from this research could inform evidence-based policies aimed at improving the HRQoL of people with cognitive impairment.

## Methods

### Data source

Our empirical analyses utilized data from the Household, Income, and Labour Dynamics in Australia (HILDA) Survey. Since 2001, the survey has been collecting yearly data from a sample of Australians who are representative of the whole country. The study monitors a cohort of almost 17,000 people over their life course, gathering data on a range of topics including family and household dynamics, employment and earnings, educational attainment, and health outcomes. The HILDA Survey used a mix of face-to-face interviews and self-completion questionnaires to obtain this information [38]. A comprehensive overview of the HILDA dataset can be found elsewhere [39].

### Study participants

Our analysis used data from two time points of the HILDA Survey: the year 2012 (wave 12) and 2016 (wave 16). These specific waves were chosen for the study because they are the only waves within the dataset that include questions specifically designed to assess cognitive impairment. The HILDA survey assessed cognitive function of the survey respondents using validated instruments, including the Symbol Digit Modalities Test (SDMT) and the Backward Digit Span Test (BDS). While the scores obtained from the BDS and SDMT provide valuable insights into specific cognitive domains (working memory and processing speed, respectively), they may not fully capture the overall cognitive profile of an individual. A limitation of these tests is the lack of established cut-off scores, which may hinder accurate diagnosis of cognitive impairment. More comprehensive neuropsychological assessments, such as the Montreal Cognitive Assessment (MoCA) [40], Mini-Mental Status Examination (MMSE) [41], or Saint Louis University Mental Status (SLUMS) examination [42], offer a nuanced and reliable evaluation of cognitive function. Due to the unavailability of data measured through these scales, we utilized the BDS and SDMT to assess cognitive impairment for this study. Evidence suggests that the BDS and SDMT have been previously used to detect cognitive impairment, particularly in people with multiple sclerosis [43, 44] and hospitalized patients [45].

**Fig. 1:**
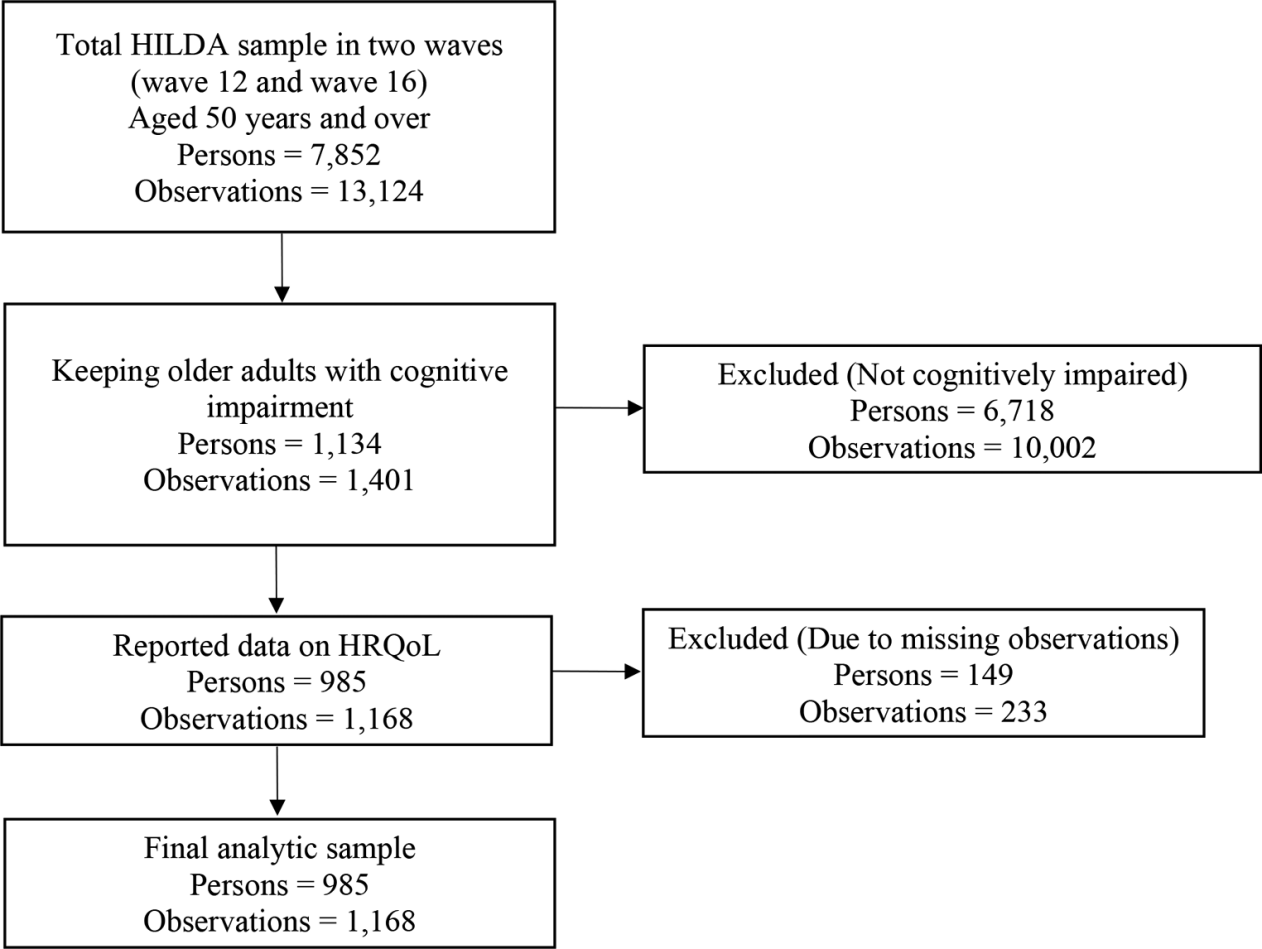
Participants’ flow in the analytic sample and missing data

The BDS cognitive evaluation exam involves individuals reciting a sequence of numbers in reverse order [45]. The BDS evaluates the cognitive capacity of working memory on a scale of 0 to 8. The SDMT is a cognitive assessment tool that measures a person’s ability to process information quickly and accurately. The SDMT requires participants to match a list of numbers with corresponding geometric shapes as quickly and accurately as possible [46]. The SDMT evaluates the cognitive function of the central brain and provides scores that range from 0 to 110. The threshold for identifying cognitive impairment in this study was informed by an established criterion. Previous research has categorized cognitive impairment based on standardized thresholds: individuals scoring ≥ 1.0 standard deviation (SD) below the mean on either the BDS or SDMT (or both) are classified as having mild cognitive impairment, while those scoring ≥ 1.5 SD below the mean on both tests are identified as having severe cognitive impairment [47–49]. This study consolidated mild and severe cognitive impairment into a single category, focusing on cognitive impairment as a unified construct. Cognitive impairment was therefore defined as scoring ≥ 1 SD below the mean on both the BDS and SDMT. This criterion aligns with empirical thresholds reported in prior studies and supports the objectives of this research. Accordingly, participants were considered cognitively impaired if they scored ≤ 3 on the BDS and ≤ 30 on the SDMT. Given that cognitive impairment primarily affects older people, our study focused on Australians aged 50 years and over. Therefore, the inclusion criteria for people in the sample were as follows: (i) being 50 years of age or older; (ii) identified as living with cognitive impairment; and (iii) having valid information on the outcome and key factors of interest. Applying these inclusion criteria resulted in an unbalanced panel of 1,168 yearly observations from 985 unique individuals. Figure 1 depicts the sample selection process and missing data analysis.

### Outcome variable

The primary outcome variable in our study was HRQoL, which we measured using the 36-item Short-Form Health Survey (SF-36). The SF-36 is a widely used and reliable instrument that assesses an individual’s physical and mental health using a standardized questionnaire [50]. The questionnaire comprises 36 items that evaluate eight specific health domains: physical functioning (PF), role physical functioning (RP), role emotional functioning (RE), social functioning (SF), mental health (MH), vitality (VT), bodily pain (BP), and general health (GH). Each dimension of the SF-36 has a theoretical range of 0 to 100, where 0 indicates the worst possible health and 100 represents the best possible health. The SF-36 data generally yields two summary measures: the physical-component summary (PCS) and the mental-component summary (MCS) [51]. The PCS and MCS were standardised by linear z-score transformations, resulting in a mean of 50 and a standard deviation of 10. The theoretical ranges of PCS and MCS scores are 4.54 to 76.09 and − 1.21 to 76.19, respectively, with higher scores indicating improved health [30].

In addition to the PCS and MCS, the SF-36 can also be used to generate the SF-6D, a health-state utility index, which is another internationally recognized measure of HRQoL [52]. The SF-6D utility index is derived from a subset of six subscales of the SF-36 (PF, RP, RE, SF, VT, and BP), with a theoretical range from 0.29 (indicating poor health) to 1 (representing optimal health) [52].

### Exposure variable

This study investigates the frequency of moderate-to-intense physical activity. To assess this, the research relies on a self-administrated question consistently employed across all waves of the HILDA Survey. This question inquires: ‘In general, how often do you participate in moderate or intensive physical activity for at least 30 minutes? Moderate physical activity will cause a slight increase in breathing and heart rate such as brisk walking.’ The participants’ replies were categorised into six pre-determined categories: ‘not at all’, ‘less than once per week’, ‘1 or 2 times per week’, ‘3 times per week’, ‘more than 3 times per week but not every day’, and ‘every day’. We simplified the physical activity categories into three groups: ‘not at all,’ ‘<1 to 3 times per week’ (by merging the groups ‘less than once per week’, ‘1 or 2 times per week’, and ‘3 times per week’), and ‘more than 3 times per week to everyday’ (by merging the groups ‘more than 3 times per week but not every day’, and ‘every day’). The categorization of physical activity in this study adheres to the established criteria of the Australian National Physical Activity Guidelines for Adults [53], demonstrating close concordance with the World Health Organization’s guidelines [54]. This methodological approach has been adopted in prior empirical research utilizing the HILDA survey data [31, 55].

### Covariates

We incorporated several individual-level socio-demographic, health-related behavioural characteristics, and health-related characteristics as covariates. The socio-economic and demographic characteristics analysed included: age (50–64 years, and ≥ 65 years), gender (male, and female), marital status (unpartnered and partnered), highest level of education (year 12 and below, professional qualifications, and university qualifications), household yearly disposable income (Quintile 1 [poorest], and Quintile 5 [richest]), participation in the labour force (employed, and unemployed or not in the labour force), Indigenous origin (non- Aboriginal or Torres Strait Islander, and Aboriginal or Torres Strait Islander), geographic residency (major city, and regional or remote area). Additionally, two health-related behavioural characteristics that can impact health outcomes were included: smoking habits (former smoker or never smoked, and currently smoking), and alcohol drinking (former drinker or never drunk, and active drinker). Furthermore, Body Mass Index (underweight, healthy weight, overweight, and obese) and disability status (no versus yes), were considered as the proxy of participants’ health-related characteristics.

### Estimation strategy

We commence our analysis by calculating descriptive statistics for the study sample. For categorical variables, frequencies and percentages are calculated to describe their distribution. For continuous variables, means and standard deviations are calculated to summarize their central tendency and variability. These descriptive statistics are calculated separately for baseline, final wave, and pooled data to provide an overview of the data across different time points. We also present a summary of participants’ SF-36 component summary scores, and SF-6D utility values according to their physical activity levels.

The outcome variables (PCS, MCS, and SF-6D) used in our study were measured on a continuous scale. Therefore, we employed a longitudinal random-effects GLS regression model to examine the relationship between physical activity and HRQoL, allowing us to identify individual variations in this association. Additionally, the random-effects structure enables us to control for unobserved individual heterogeneity and potential confounders, thereby enhancing the reliability of our findings.

The random-effects GLS regression can be expressed in the following functional form:
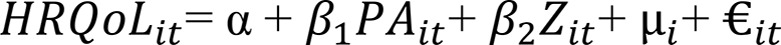


HRQoL denotes the three outcome variables: PCS, MCS and SF-6D. The level of physical activity, PA, is an exposure variable. $\:{Z}_{it}$ represents the vector consisting of time-varying and time-invariant control variables. The model parameter of interest to be estimated is denoted as $\>{\beta _1}$, and $\>{\beta _2}$ indicates the vector of coefficients while α is the model’s grand intercept. The analysis considers two parts of the error: individual-specific components, $\:{\upmu\:}$, that stays the same over time, and time and person-specific error, $\:{\text{€}}_{it}$, which is assumed to be uncorrelated with the independent variables.

Statistical significance was determined using a p-value threshold of 0.05. Lower p-values (< 0.01 and < 0.001) were reported to indicate stronger evidence of significance. Stata version 17.0 (StataCorp LLC, College Station, TX: USA) was employed for all statistical analyses.

## Results

Table 1 displays the socio-economic, demographic, health-related behavioural, and health-related characteristics of the analytic sample at baseline, final wave, and pooled across waves. In the pooled data, most participants were older adults, with 79% aged 65 years or older, 48% were female, and 53% were partnered. Among the study sample, 8% had a university degree, about 16% were employed, just below 2% identified as Aboriginal and Torres Strait Islander, 57% resided in major cities, 11% were smokers, 66% drank alcohol, 26% were obese, and 65% had a disability.

**Table 1:** Distribution of analytic sample (socio-economic and demographic, health-related behavioural characteristics, and health-related characteristics: baseline, final, and pooled across all waves)

Variables	Baseline Wave (2012)		Final Wave (2016)		Pooled data (2012–2016)	
	*n*	%	*n*	%	*n*	%
**Socio-economic, and demographic characteristics**						
**Age group**						
*50–64 years*	131	20.70	116	21.68	247	21.15
*65 years and over*	502	79.30	419	78.32	921	78.85
**Gender**						
*Male*	325	51.34	286	53.46	611	52.31
*Female*	308	48.66	249	46.54	557	47.69
**Marital status**						
*Unpartnered*	306	48.34	246	45.98	552	47.26
*Partnered*	327	51.66	289	54.02	616	52.74
**Highest level of education**						
*Year 12 and below*	422	66.67	327	61.12	749	64.13
*Professional qualifications*	167	26.38	161	30.09	328	28.08
*University qualifications*	44	6.95	47	8.79	91	7.79
**Household yearly disposable income (Quintile)**						
*Quintile 1 (poorest)*	168	26.54	66	12.34	234	20.03
*Quintile 2*	134	21.17	100	18.69	234	20.03
*Quintile 3*	94	14.85	139	25.98	233	19.95
*Quintile 4*	118	18.64	117	21.87	235	20.12
*Quintile 5 (richest)*	119	18.80	113	21.12	232	19.86
**Participation in labour force**						
*Employed*	98	15.48	85	15.89	183	15.67
*Unemployed or not in the labour force*	535	84.52	450	84.11	985	84.33
**Indigenous origin**						
*Non-Aboriginal or Torres Strait Islander*	624	98.58	524	97.94	1,148	98.29
*Aboriginal or Torres Strait Islander*	9	1.42	11	2.06	20	1.71
**Geographic residency**						
*Major cities*	356	56.24	309	57.76	665	56.93
*Regional/remote*	277	43.76	226	42.24	503	43.07
**Health-related behavioural characteristics**						
**Smoking habits**						
*Former smoker/never smoked*	557	87.99	479	89.53	1,036	88.70
*Currently smoking*	76	12.01	56	10.47	132	11.30
**Alcohol drinking**						
*Former drinker or never drunk*	195	30.81	199	37.20	394	33.73
*Active drinker*	438	69.19	336	62.80	774	66.27
**Health-related characteristics**						
**Body Mass Index (BMI)**						
*Underweight*	24	3.79	13	2.43	37	3.17
*Healthy weight*	225	35.55	178	33.27	403	34.50
*Overweight*	233	36.81	189	35.33	422	36.13
*Obesity*	151	23.85	155	28.97	306	26.20
**Disability status**						
*No*	229	36.18	183	34.21	412	35.27
*Yes*	404	63.82	352	65.79	756	64.73

Notes (1) The pooled study comprised a total of 1,168 person-year observations from 985 distinct individuals. (2) The OECD-modified equivalency scale was used to calculate the equivalised yearly household income, which was then divided into quintiles

Table 2 displays the summary statistics of key variables, including PCS, MCS, SF-6D utility value, eight dimensions of the SF-36, and levels of physical activity among the study participants. In the pooled data, the mean PCS, MCS, and SF-6D values of the study participants were 37.86, 48.15 and 0.67, respectively. The mean score of the SF-36’s eight dimensions were as follows: PF (56.47), RP (46.24), RE (64.14), SF (69.44), MH (71.88), VT (54.27), BP (56.50), and GH (54.46). Regarding physical activity, the pooled data also showed that approximately 30% of the participants do not perform in any physical activity, around 44% participated in moderate or intense physical activity < 1 to 3 times per week, and around 26% engaged in moderate or intense physical activity more than 3 times per week to every day.

**Table 2:** Summary statistics: subjective health scores, and level of physical activity

Characteristics	Baseline Wave (2012)		Final Wave (2016)		Pooled data (2012–2016)	
	*n*	% /mean (SD)	*n*	% /mean (SD)	*n*	% /mean (SD)
**SF-36 domain scores**						
*Physical functioning*	633	56.76 (29.27)	535	56.11 (29.88)	1,168	56.47 (29.54)
*Role physical*	633	46.87 (44.88)	535	45.48 (45.27)	1,168	46.24 (45.04)
*Role emotional*	633	64.01 (43.38)	535	64.30 (43.40)	1,168	64.14 (43.37)
*Social functioning*	633	70.97 (27.17)	535	67.64 (28.36)	1,168	69.44 (27.76)
*Mental health*	633	72.22 (18.43)	535	71.48 (17.80)	1,168	71.88 (18.14)
*Vitality*	633	54.80 (21.44)	535	53.64 (21.08)	1,168	54.27 (21.28)
*Bodily pain*	633	57.06 (26.37)	535	55.84 (26.75)	1,168	56.50 (26.54)
*General health*	633	55.31 (23.72)	535	53.46 (22.85)	1,168	54.46 (23.33)
**SF-36 component summary score**						
*PCS*	633	38.09 (11.91)	535	37.59 (12.11)	1,168	37.86 (12.00)
*MCS*	633	48.43 (10.67)	535	47.82 (10.57)	1,168	48.15 (10.62)
*SF-6D utility value*	633	0.67 (0.13)	535	0.68 (0.13)	1,168	0.67 (0.13)
**Levels of physical activity**						
*Not at all*	184	29.07	170	31.78	354	30.31
*< 1 to 3 times per week*	277	43.76	233	43.55	510	43.66
*More than 3 times per week to everyday*	172	27.17	132	24.67	304	26.03

Notes (1) The pooled study comprised a total of 1,168 person-year observations from 985 distinct individuals. (2) PCS = physical component summary, MCS = mental component summary, and SF-6D = Short-Form Six-Dimension health utility index

**Fig. 2:**
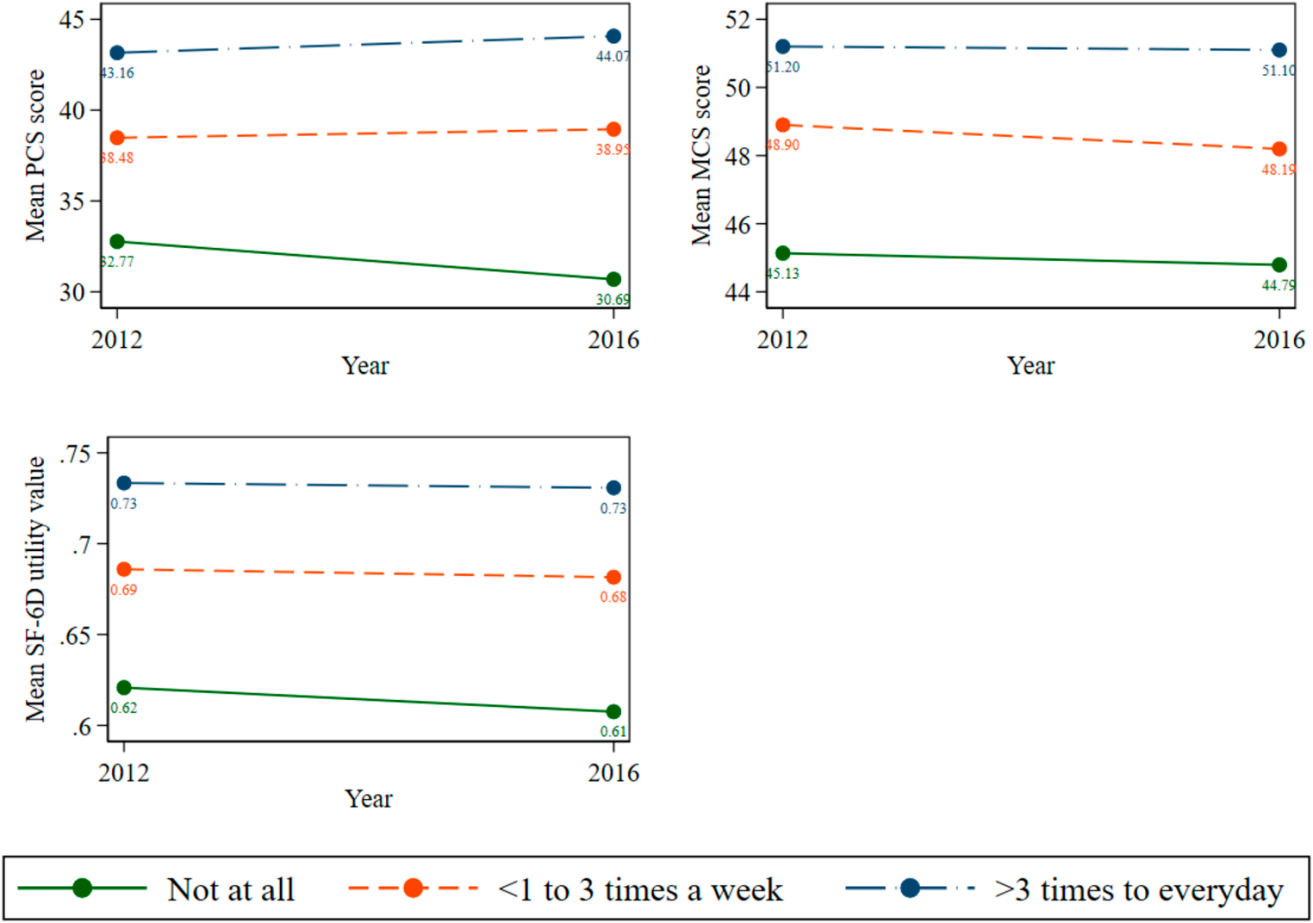
Mean PCS, MCS, and SF-6D utility values by the status of level of physical activities. Note: 1. Abbreviation: PCS = physical component summary, MCS = mental component summary, and SF-6D = Short-Form Six-Dimension health utility index

Figure 2 depicts the mean PCS scores, MCS scores, and SF-6D utility value among older Australians with cognitive impairment based on their physical activity levels. The results show that those who were physically inactive exhibited lower PCS, MCS, and SF-6D scores compared to their counterparts. For instance, in wave 16, people who never engaged in physical activity had the lowest scores (PCS = 30.69, MCS = 44.79, SF-6D = 0.61), followed by those engaged in moderate or intense physical activity < 1–3 times per week (PCS = 38.95, MCS = 48.19, SF-6D = 0.68), with the highest scores observed in those engaged in moderate or intense physical activity more than 3 times per week to every day (PCS = 44.07, MCS = 51.10, SF-6D = 0.73).

Table 3 presents the results obtained from the random-effects GLS regression models. We found that older Australians with cognitive impairment who engaged in moderate or intense physical activity for at least 30 min had higher PCS, MCS and SF-6D values compared to those who were physically inactive. For example, model 1 demonstrated that people who engaged in moderate or intense physical activity < 1–3 times per week, and more than 3 times per week to everyday had higher PCS scores 4.41 (β = 4.41, SE = 0.68) and 7.28 (β = 7.28, SE = 0.82), respectively, compared to those who were physically inactive. Similarly, model 2 revealed that those who engage in moderate or intense physical activity < 1–3 times per week, and more than 3 times per week to everyday had higher MCS scores 2.55 (β = 2.55, SE = 0.74) and 4.10 (β = 4.10, SE = 0.84), respectively, relative to physically inactive people. Additionally, from model 3, we found that participants engaged in moderate or intense physical activity < 1–3 times per week, and more than 3 times per week to everyday had greater SF-6D utility values 0.05 (β = 0.05, SE = 0.007) and 0.07 (β = 0.07, SE = 0.009), respectively, in comparison to those who did not engage in any physical activity.

**Table 3:** Abridged results from random-effects GLS regression models of HRQoL (MCS, PCS and SF-6D), pooled analysis

Variables	Model 1	Model 2	Model 3
	PCS	MCS	SF-6D
	Coefficient (SE), *P*-value	Coefficient (SE), *P*-value	Coefficient (SE), *P*-value
**Levels of physical activity**			
*Not at all (ref)*			
*< 1 to 3 times per week*	4.41 (0.68), 0.001	2.55 (0.74), 0.001	0.05 (0.007), 0.001
*More than 3 times per week to everyday*	7.28 (0.82), 0.001	4.10 (0.84), 0.001	0.07 (0.009), 0.001

Notes (1) The sample size is 985 individuals and 1,168 observations. (2) All models were adjusted for age, gender, marital status, highest education level of education, household yearly disposable income, participation in the labour force, Indigenous origin, geographic residency, smoking habits, alcohol drinking, BMI and disability status. (3) PCS = physical component summary, MCS = mental component summary, and SF-6D = Short-Form Six-Dimension health utility index. (4) Ref means reference category. (5) Cluster-robust standard errors (SE) are reported in the parenthesis

### Robustness check

We also fitted random-effects generalised estimating equation (GEE) regression model as part of the sensitivity analysis. The model initially reported in Table 3 was re-assessed and presented in Appendix Table A1 in the online supplemental material. The findings of the sensitivity analysis closely matched the baseline values for all the factors of interest. We found that older Australians with cognitive impairment who engaged in moderate or intense physical activity had higher PCS, MCS and SF-6D values compared to those who were physically inactive. For example, participants engaged in moderate or intense physical activity for more than 3 times per week to everyday had greater PCS (β = 7.46, SE = 0.76), MCS (β = 4.61, SE = 0.81) and SF-6D (β = 0.08, SE = 0.008) scores, in comparison to those who did not engage in physical activity.

### Sensitivity analysis

We conducted a missing data analysis, as presented in Appendix Table A2. With the exception of Indigenous origin, SF-6D utility score, and BMI, the proportion of missing observations for most variables was less than 5%. To address the missing data, we applied the last value carry forward imputation technique. After imputation, we applied the random-effects GLS technique and compared the results with the estimates obtained from the complete case analysis (Table 3). The regression results from the imputed data were consistent in direction with the baseline regression findings. However, the magnitudes of the physical activity estimates varied slightly across the HRQoL measures. For example, participants engaged in moderate or intense physical activity for more than 3 times per week to everyday had greater PCS (β = 7.53, SE = 0.79), MCS (β = 3.97, SE = 0.83) and SF-6D (β = 0.07, SE = 0.009) scores, in comparison to those who did not engage in physical activity. The imputed regression analysis results are detailed in Appendix Table A3.

### Heterogenous effect

To further explore the relationship between physical activity and HRQoL, we ran a series of random-effects Generalized Least Squares (GLS) regression models. These models were used to investigate how the association between physical activity and HRQoL might vary across different age and gender subgroups within the study population. The results indicated that, across both age groups (50–64 years and 65 years and older), people who participated in any form of moderate or intense physical activity for at least 30 min had significantly higher PCS, MCS, and SF-6D values compared to those who were physically inactive (see Table 4). For instance, among participants aged 65 years and over, those engaging in moderate or intense physical activity more than 3 times per week to everyday had significantly higher scores in PCS (β = 7.41, SE = 0.91), MCS (β = 4.51, SE = 0.96), and SF-6D (β = 0.07, SE = 0.011) compared to those who did not engage in physical activity (Models 4, 5 and 6 in Table 4). Similarly, irrespective of gender, individuals participating in any moderate or intense physical activity showed higher PCS, MCS, and SF-6D scores than those who were inactive (see Table 5). For example, female participants who engaged in moderate or intense physical activity more than 3 times per week to daily had greater PCS (β = 7.16, SE = 1.21), MCS (β = 2.10, SE = 1.23), and SF-6D (β = 0.05, SE = 0.01) scores compared to their inactive counterparts (Models 4, 5 and 6 in Table 5).

**Table 4:** Abridged results from random-effects GLS regression models of HRQoL (MCS, PCS and SF-6D) by age

Variables	Age (50–64 years)			Age (65 years and above)		
	Model 1	Model 2	Model 3	Model 4	Model 5	Model 6
	PCS	MCS	SF-6D	PCS	MCS	SF-6D
	Coefficient (SE), P-value	Coefficient (SE), P-value	Coefficient (SE), P-value	Coefficient (SE), P-value	Coefficient (SE), P-value	Coefficient (SE), P-value
**Levels of physical activity**						
*Not at all (ref)*						
*< 1 to 3 times per week*	3.62 (1.69), 0.03	1.18 (1.77), 0.50	0.04 (0.01), 0.03	4.36 (0.75), 0.001	2.94 (0.82), 0.001	0.04 (0.008), 0.001
*More than 3 times per week to everyday*	6.67 (1.78), 0.001	3.09 (1.88), 0.10	0.06 (0.02), 0.01	7.41 (0.91), 0.001	4.51 (0.96), 0.001	0.07 (0.011), 0.001

Note (1) All models were adjusted for age, gender, marital status, highest education level of education, household yearly disposable income, participation in the labour force, Indigenous origin, geographic residency, smoking habits, alcohol drinking, BMI and disability status. (2) PCS = physical component summary, MCS = mental component summary, and SF-6D = Short-Form Six-Dimension health utility index. (3) Ref means reference category. (4) Cluster-robust standard errors (SE) are reported in the parenthesis

**Table 5:** Abridged results from random-effects GLS regression models of HRQoL (MCS, PCS and SF-6D) by gender

Variables	Male			Female		
	Model 1	Model 2	Model 3	Model 4	Model 5	Model 6
	PCS	MCS	SF-6D	PCS	MCS	SF-6D
	Coefficient (SE), *P*-value	Coefficient (SE), *P*-value	Coefficient (SE), *P*-value	Coefficient (SE), *P*-value	Coefficient (SE), *P*-value	Coefficient (SE), *P*-value
**Levels of physical activity**						
*Not at all (ref)*						
*< 1 to 3 times per week*	3.02 (0.97), 0.01	3.43 (1.06), 0.001	0.04 (0.01), 0.001	5.70 (0.95), 0.001	1.93 (0.98), 0.05	0.04 (0.01), 0.001
*More than 3 times per week to everyday*	6.90 (1.11), 0.001	6.11 (1.09), 0.001	0.08 (0.01), 0.001	7.16 (1.21), 0.001	2.10 (1.23), 0.08	0.05 (0.01), 0.001

Note (1) All models were adjusted for age, gender, marital status, highest education level of education, household yearly disposable income, participation in the labour force, Indigenous origin, geographic residency, smoking habits, alcohol drinking, BMI and disability status. (2) PCS = physical component summary, MCS = mental component summary, and SF-6D = Short-Form Six-Dimension health utility index. (3) Ref means reference category. (4) Cluster-robust standard errors (SE) are reported in the parenthesis

## Discussion

Our research sought to evaluate the relationship between physical activity and HRQoL among older Australians living with cognitive impairment. We employed a mix of preference-based (SF-6D) and non-preference (SF-36 component summaries) measures to assess HRQoL. Our analysis, utilizing random-effects Generalized Least Squares (GLS) modelling, demonstrated that physical activity acts as a protective factor for HRQoL. We discovered that older Australians with cognitive impairment engaging in moderate or intense physical activity for at least 30 min had substantially greater PCS, MCS, and SF-6D utility values than their physically inactive counterparts.

Our results are consistent with earlier research conducted across various countries among general populations (e.g., had no cognitive impairment) and found a positive association between physical activity and HRQoL [24–27]. An earlier study also reported that consistent physical activity levels, including gradual increases in activity frequency, were linked to preserving or enhancing both physical and mental HRQoL in community-dwelling older adults [28]. Furthermore, two Australian longitudinal studies provided evidence that a higher frequency of moderate-to-vigorous intense physical activity was linked to an improved HRQoL [30, 31].

Our findings also align with one RCT study that demonstrated the benefits of aerobic exercise training in reducing the decline in HRQoL in older adults with mild cognitive impairment [57]. However, another RCT study did not find a significant positive effect of walking on quality of life within a similar cohort [56]. The observed discrepancies between our findings and those of prior studies may be explained by variations in research design, physical activity assessment methods, study populations, and the specific criteria used to diagnose cognitive impairment.

The relationship between physical activity and HRQoL may be explained by its positive effects on functional capacity and physical health, which promote a greater sense of independence and well-being [21]. These improvements are likely to enhance the physical aspects of HRQoL. Moreover, physical activity may benefit people with cognitive impairment by improving sleep quality and reducing depressive symptoms, thereby positively influencing HRQoL [57]. Additionally, physical activity may enhance HRQoL by affecting mood-related brain chemicals, such as neurotransmitters and endorphins [58, 59]. Regular physical activity also improves physical fitness, functionality, and a sense of control [26, 60, 61], and is associated with increased mental stimulation and better psychological health [26]. Social interaction may also be an important factor in explaining the observed relationship between physical activity levels and HRQoL. Many forms of physical activity, such as group exercise classes, walking clubs, or team sports, provide opportunities for social engagement, which has a positive impact on mental health and psychological well-being. Social interactions fostered through physical activity can reduce feelings of isolation and loneliness, particularly among individuals with cognitive impairment, thereby contribute to the improvement of the mental aspects of HRQoL [62, 63]. Furthermore, a history of regular physical activity may have a protective influence on future physical activity levels, HRQoL, and cognitive impairment. Engaging in physical activity throughout earlier life stages can have long-term benefits. These benefits, likely stemming from the cumulative effects of improved muscle strength, cardiovascular health, and neural plasticity, may contribute to better physical and cognitive health in later years [64, 65]. This cumulative effect of early-life physical activity on physical and cognitive health may create a virtuous cycle. Improved physical and cognitive health can motivate continued engagement in physical activity, which in turn can further enhance HRQoL and create a positive feedback loop that supports overall well-being.

### Strengths, limitations, and avenues for further research

Our study’s strengths include its use of a comprehensive longitudinal design. To the best of our knowledge, this is the first observational study to examine the association between physical activity and HRQoL among people with cognitive impairment. The use of a validated instrument to measure HRQoL and cognitive impairment ensures the reliability of our findings. However, our study has some limitations that need to be mentioned. Firstly, the HILDA Survey’s physical activity frequency measure may not have accurately captured the exact amount of time participants spent on physical activities, which could affect the precision of our results. Secondly, the reliance on self-reported data for physical activity and other covariates may introduce potential biases. Social desirability bias, characterised by the inclination of individuals to overstate their physical activity levels or underreport health limitations, is a significant concern. Recall bias, especially among older adults and those with cognitive impairments, can also distort the accuracy of reported physical activity engagement and HRQoL experiences. These biases may lead to either under- or overestimation of the true association between physical activity and HRQoL. Third, the absence of standardized cut-off scores for the SDMT and BDS limits their ability to accurately define and comprehensively assess the full range of cognitive impairment. Furthermore, while these tests provide valuable insights into specific cognitive domains, they may not adequately capture memory deficits, a core feature of cognitive impairment and dementia. This limitation could have potentially underestimated the prevalence of cognitive impairment in this study. The inclusion of memory-focused assessments, such as the Montreal Cognitive Assessment, Mini-Mental State Examination, or Saint Louis University Mental Status test, could have provided a more targeted approach and improved comparability with findings from other studies. Finally, the analysis did not account for potential confounding factors such as diet, stress levels, and comorbid conditions. These factors can significantly influence both physical activity levels and HRQoL. The unavailability of data on these factors in the HILDA Survey represents a major limitation. By not accounting for these factors, the study may not precisely estimated the relationship between physical activity and HRQoL among people with cognitive impairment. This may lead to confounding bias, distorting the observed relationship between physical activity and HRQoL due to the influence of these unobserved variables. This could result in spurious associations.

Future research could explore the economic implications of physical activity in people with cognitive impairment, including cost savings, work productivity, and reduction of overall health and economic burdens on the healthcare system. These research would provide valuable insights for policymakers and healthcare providers, guiding strategies and investments in initiatives concerning physical activity.

### Implications for policy and practice

The findings of this research can be directly utilized by healthcare professionals who work with older adults with cognitive impairment to develop and implement more effective health promotion interventions that emphasize the importance of physical activity. In 2018, Australia became one of the pioneering nations in developing national physical activity guidelines tailored to older adults with cognitive impairment [66]. A narrative review further supported these guidelines, providing evidence that the recommendations for older adults could be adapted to meet the needs of individuals with cognitive impairment [67]. This conclusion was reached after comparing these guidelines with similar Canadian guidelines. Evidence suggests that older Australians today place a higher value on information and advice from healthcare professionals compared to other sources, highlighting their critical role in adopting recommendations [67]. By offering tailored guidance and support, healthcare professionals can assist individuals in achieving the recommended physical activity levels, which can lead to improved well-being. Our findings highlight the necessity of including physical activity in comprehensive care plans through bulk-billed GP services for older adults with cognitive impairment.

## Conclusions

Our research provides substantial evidence of the positive relationship between physical activity and HRQoL in older people with cognitive impairment. Using longitudinal data from the HILDA Survey, we found that engaging in moderate to intense physical activity for at least 30 min is associated with improved HRQoL. These results highlight the significance of integrating physical activity into holistic strategies for enhancing the general well-being of older Australians living with cognitive impairment. Health education and promotion initiatives must be implemented across all demographics to promote physical activity, especially amongst those with cognitive impairments. The SF-6D utility values derived from our research may serve as essential inputs for the forthcoming economic evaluation of intervention concerning physical activity. Thereby, our study findings will help policy makers to identify the cost-effective interventions concerning physical activity aimed at improving the health and well-being of older adults. Further research is needed to investigate the enduring advantages of physical activity on HRQoL in adults with cognitive impairment and to create tailored therapy that encourage physical activity.

## Electronic supplementary material

Below is the link to the electronic supplementary material.

Supplementary Material 1

## Data Availability

The data were obtained from the Melbourne Institute of Applied Economic and Social Research (https://melbourneinstitute.unimelb.edu.au/). Though the information is not openly available, appropriately qualified researchers can access the data after following their protocols and meeting their requirements. Their contact address is Melbourne Institute of Applied Economic and Social Research, the University of Melbourne, VIC 3010, Australia.
