# Supplementary material for: Staying active, staying sharp: the relationship between physical activity and health-related quality of life for people living with cognitive impairment
Source: Qual Life Res. 2025 Feb 8;34(5):1443–56. doi: 10.1007/s11136-025-03910-5 (PMC12064627; doi:10.1007/s11136-025-03910-5)
Supplement: Supplementary file 1 — Supplementary Material 1 [file 11136_2025_3910_MOESM1_ESM.docx]

**Appendix**

STROBE Statement—Checklist of items that should be included in reports of ***observational studies***

|  | **Item No** | **Recommendation** | **Page No** |
| --- | --- | --- | --- |
| **Title and abstract** | 1 | (*a*) Indicate the study’s design with a commonly used term in the title or the abstract | 1 |
|  |  | (*b*) Provide in the abstract an informative and balanced summary of what was done and what was found | 1 |
| **Introduction** | | | |
| Background/rationale | 2 | Explain the scientific background and rationale for the investigation being reported | 2 |
| Objectives | 3 | State specific objectives, including any prespecified hypotheses | 4 |
| **Methods** | | | |
| Study design | 4 | Present key elements of study design early in the paper | 4 |
| Setting | 5 | Describe the setting, locations, and relevant dates, including periods of recruitment, exposure, follow-up, and data collection | 4 |
| Participants | 6 | (*a*) Give the eligibility criteria, and the sources and methods of selection of participants | 4 |
| Variables | 7 | Clearly define all outcomes, exposures, predictors, potential confounders, and effect modifiers. Give diagnostic criteria, if applicable | 6-8 |
| Data sources/ measurement | 8* | For each variable of interest, give sources of data and details of methods of assessment (measurement). Describe comparability of assessment methods if there is more than one group | 4 |
| Bias | 9 | Describe any efforts to address potential sources of bias | N/A |
| Study size | 10 | Explain how the study size was arrived at | 5-6 |
| Quantitative variables | 11 | Explain how quantitative variables were handled in the analyses. If applicable, describe which groupings were chosen and why | N/A |
| Statistical methods | 12 | (*a*) Describe all statistical methods, including those used to control for confounding | 8-9 |
|  |  | (*b*) Describe any methods used to examine subgroups and interactions | 8-9 |
|  |  | (*c*) Explain how missing data were addressed | 16 |
|  |  | (*d*) If applicable, describe analytical methods taking account of sampling strategy | N/A |
|  |  | (*e*) Describe any sensitivity analyses | 16 |
| **Results** | | | |
| Participants | 13* | (a) Report numbers of individuals at each stage of study—eg numbers potentially eligible, examined for eligibility, confirmed eligible, included in the study, completing follow-up, and analysed | 4 |
|  |  | (b) Give reasons for non-participation at each stage | 4-5 |
|  |  | (c) Consider use of a flow diagram | 6 |
| Descriptive data | 14* | (a) Give characteristics of study participants (eg demographic, clinical, social) and information on exposures and potential confounders | 11-13 |
|  |  | (b) Indicate number of participants with missing data for each variable of interest | Appendix Table A2 |
| Outcome data | 15* | Report numbers of outcome events or summary measures | 13-14 |
| Main results | 16 | (*a*) Give unadjusted estimates and, if applicable, confounder-adjusted estimates and their precision (eg, 95% confidence interval). Make clear which confounders were adjusted for and why they were included | 15-20 |
|  |  | (*b*) Report category boundaries when continuous variables were categorized | 13 |
|  |  | (*c*) If relevant, consider translating estimates of relative risk into absolute risk for a meaningful time period | N/A |
| Other analyses | 17 | Report other analyses done—eg analyses of subgroups and interactions, and sensitivity analyses | 15-16 |
| **Discussion** | | | |
| Key results | 18 | Summarise key results with reference to study objectives | 20 |
| Limitations | 19 | Discuss limitations of the study, taking into account sources of potential bias or imprecision. Discuss both direction and magnitude of any potential bias | 21-22 |
| Interpretation | 20 | Give a cautious overall interpretation of results considering objectives, limitations, multiplicity of analyses, results from similar studies, and other relevant evidence | 20-21 |
| Generalisability | 21 | Discuss the generalisability (external validity) of the study results | 20 |
|  | | | |
| Funding | 22 | Give the source of funding and the role of the funders for the present study and, if applicable, for the original study on which the present article is based | 30 |

*Give information separately for exposed and unexposed groups.

**Note:** An Explanation and Elaboration article discusses each checklist item and gives methodological background and published examples of transparent reporting. The STROBE checklist is best used in conjunction with this article (freely available on the Web sites of PLoS Medicine at http://www.plosmedicine.org/, Annals of Internal Medicine at http://www.annals.org/, and Epidemiology at http://www.epidem.com/). Information on the STROBE Initiative is available at www.strobe-statement.org.

**Appendix Table A1: Abridged results from random effect GEE regression models of HRQoL (MCS, PCS and SF-6D), pooled analysis**

|  | **Model 1** | **Model 2** | **Model 3** |
| --- | --- | --- | --- |
|  | **PCS** | **MCS** | **SF-6D** |
|  | Coefficient (SE), P-value | Coefficient (SE), P-value | Coefficient (SE), P-value |
| **Level of physical activity** |  |  |  |
| *Not at all (ref)* |  |  |  |
| *1 to 3 times a week* | 4.66 (0.66), 0.001 | 2.63 (0.70), 0.001 | 0.04 (0.007), 0.001 |
| *More than 3 times a week to everyday* | 7.46 (0.76), 0.001 | 4.61 (0.81), 0.001 | 0.08 (0.008), 0.001 |

Note: 1. The sample size is 985 individuals and 1,168 observations. 2. All models were adjusted for age, gender, marital status, highest education level of education, household yearly disposable income, participation in the labour force, Indigenous origin, geographic residency, smoking habits, alcohol drinking, BMI and disability status. 3. PCS=physical component summary, MCS=mental component summary, and SF-6D=Short-Form Six-Dimension health utility index. 4. Ref means reference category. 5. Cluster-robust standard errors (SE) are reported in the parenthesis.

**Appendix Table A2: Missing observation analysis**

| **Variable** | **Missing observation** | **Total observation** | **Percent Missing** |
| --- | --- | --- | --- |
|  |  |  |  |
| SF-6D utility score | 194 | 1,401 | 13.85 |
| Backward digit score | 0 | 1,401 | 0 |
| Symbol-digit modality score | 0 | 1,401 | 0 |
| Physical functioning | 51 | 1,401 | 3.64 |
| Role physical | 100 | 1,401 | 7.14 |
| Role emotional | 100 | 1,401 | 7.14 |
| Social functioning | 8 | 1,401 | 0.57 |
| Mental health | 26 | 1,401 | 1.86 |
| Vitality | 26 | 1,401 | 1.86 |
| Bodily pain | 20 | 1,401 | 1.43 |
| General Health | 65 | 1,401 | 4.64 |
| Age | 0 | 1,401 | 0 |
| Gender | 0 | 1,401 | 0 |
| Marital status | 0 | 1,401 | 0 |
| Highest level of education | 0 | 1,401 | 0 |
| Participation in labour force | 0 | 1,401 | 0 |
| Indigenous origin | 424 | 1,401 | 30.26 |
| Geographic residency | 0 | 1,401 | 0 |
| Body Mass Index (BMI) | 141 | 1,401 | 10.06 |
| Disability status | 1 | 1,401 | 0.07 |
| Smoking habits | 52 | 1,401 | 3.71 |
| Alcohol drinking | 0 | 1,401 | 0 |
| Levels of physical activity | 21 | 1,401 | 1.5 |

**Appendix Table A3: Abridge results from random effect GLS regression models of HRQoL (MCS, PCS and SF-6D) (with imputed value)**

|  | **Model 1** | **Model 2** | **Model 3** |
| --- | --- | --- | --- |
|  | **PCS** | **MCS** | **SF-6D** |
|  | Coefficient (SE), P-value | Coefficient (SE), P-value | Coefficient (SE), P-value |
| Level of physical activity |  |  |  |
| Not at all (ref) |  |  |  |
| 1 to 3 times per week | 4.82 (0.65), 0.001 | 2.38 (0.72),0.001 | 0.04 (0.008), 0.001 |
| More than 3 times per week to everyday | 7.53 (0.79), 0.001 | 3.97 (0.83), 0.001 | 0.07 (0.009), 0.001 |

1. All models were adjusted for age, gender, marital status, highest education level of education, household yearly disposable income, participation in the labour force, Indigenous origin, geographic residency, smoking habits, alcohol drinking, BMI and disability status. 3. PCS=physical component summary, MCS=mental component summary, and SF-6D=Short-Form Six-Dimension health utility index. 4. Ref means reference category. 5. Cluster-robust standard errors (SE) are reported in the parenthesis.
